# Association of depressive symptoms with retirement in Chinese employees: evidence from national longitudinal surveys from 2011 to 2018

**DOI:** 10.1186/s12889-023-15971-7

**Published:** 2023-05-26

**Authors:** Fenglin Xu, Jingmin Yuan, Hongxia Wu

**Affiliations:** 1 Department of Nursing, Hubei College of Chinese Medicine, Jingzhou, China; 2 Health Science Center, Yangtze University, Jingzhou, China; 3 Department of Respiratory and Critical Care Medicine, Shanxi Provincial People’s Hospital, Taiyuan, China

**Keywords:** Retirement, Depressive symptom, China, Older adults, CHARLS

## Abstract

**Background:**

The relationship between depressive symptoms and retirement remains controversial. Thus, we aimed to explore the effect of retirement on individuals' depressive symptoms in Chinese employees.

**Methods:**

In this panel data analysis, a data set from China Health and Retirement Longitudinal Study (CHARLS) in 2011, 2013, 2015 and 2018 was adopted with a total of 1390 employees aged ≥ 45-years-old who had complete follow-up for the four waves. Random-effects logistic regression was used to examine the associations between retirement and depressive symptoms.

**Results:**

After adjusting several socio-demographic variables, retirement still increases the risk of depressive symptoms in the retirees (odds ratio 1.5, 95% CI 1.14–1.97). Through subgroup analysis, we found that people who are male, with lower education level, married, living in rural areas, suffering from chronic diseases, and those who do not participate in social activities are more likely to experience depression after retirement.

**Conclusions:**

Retirement can increase the depression risk of Chinese employees. It is necessary to formulate relevant supporting policies to reduce the risk of depression.

**Supplementary Information:**

The online version contains supplementary material available at 10.1186/s12889-023-15971-7.

## Introduction

According to the latest data from the seventh national census, the proportion of people aged 60 and over in the total population in China is 18.7 percent, which is 5.44 percentage points higher than the 2010 level in the last census [1]. The increase in life expectancy and the decrease in fertility rates have led to a decrease in the labor force, which means that the “demographic bonus” is disappearing. With the retirement of the "baby boomers" born in the 1950s and 1960s, the problem of getting old before getting rich in China has become increasingly serious. An effective response is to raise the statutory retirement age [2].

Depression is highly prevalent in elderly populations. Risk factors for depression in the older population include age-related and disease-related processes, as well as psychosocial adversity. Psychosocial adversity-economic impoverishment, disability, isolation, relocation, caregiving, and bereavement-contributes to physiological changes, further increasing susceptibility to depression or triggering depression in already vulnerable elderly individuals [3]. Retirement is an important turning point in an individual’s life and thereby has potential mental health consequences, as it can disrupt social support networks but may also terminate exposure to stressful working conditions [4]. In 2017, the Chinese government proposed a Healthy China Strategy to promote the transformation of the demographic bonus to a “health bonus”, improve labor life, and reduce the negative impact of population aging on the labor structure [5]. Given that older individuals are more susceptible to physical and psychological problems [5], exploring the causal relationship between retirement and health is helpful to the formulation and optimization of retirement policy.

Although many researchers from different countries have explored the association between retirement and depression, the results are not consistent. Michael found that retirement may affect health through changing health-related behaviors,.such as smoking and exercise [6]. Relief from the stresses and strains of work, increased sleep time and more frequent physical activity appear to be key mechanisms by which retirement affects health [7]. Behncke et al. found retirement significantly increases the risk of being diagnosed with a chronic condition, and raises the risk of a severe cardiovascular disease and cancer [8]. Peristera et al. believe that different people have different reactions to retirement, and the trajectory of the impact of retirement on the mental health of the population is also heterogeneous [4]. The multilevel model of retirement suggested that one’s behaviors and actions throughout the retirement process are not only influenced by the individual-level variables but also shaped by the larger context of their retirement (i.e., the macro- and mesolevel variables) [9]. In China, there is very little evidence of the relationship between retirement and depression. We present the first study from China that uses panel survey data for the period 2011–18 to examine retirement effect on depression.

## Methods

### Study design and data sources

In this panel data analysis, we used data from the four waves of China Health and Retirement Longitudinal Study (CHARLS), which collects high-quality data with a structured questionnaire from a nationally representative sample of Chinese residents aged 45 years and older, selected using multistage stratified probability-proportionate-to-size sampling. The data included individual weighting variables to ensure that the survey sample was nationally representative. The baseline survey was conducted between June 2011 and March 2012, which included 17,708 individual respondents from 450 villages within 28 provinces. All participants were assessed by one-to-one interviews. Participants were followed up in 2013, 2015 and 2018. A detailed description of the objectives and methods of CHARLS has been reported elsewhere [10]. CHARLS was approved by the Biomedical Ethics Review Committee of Peking University, Beijing, China (IRB00001052–11,015). All participants provided written informed consent.

**Fig. 1:**
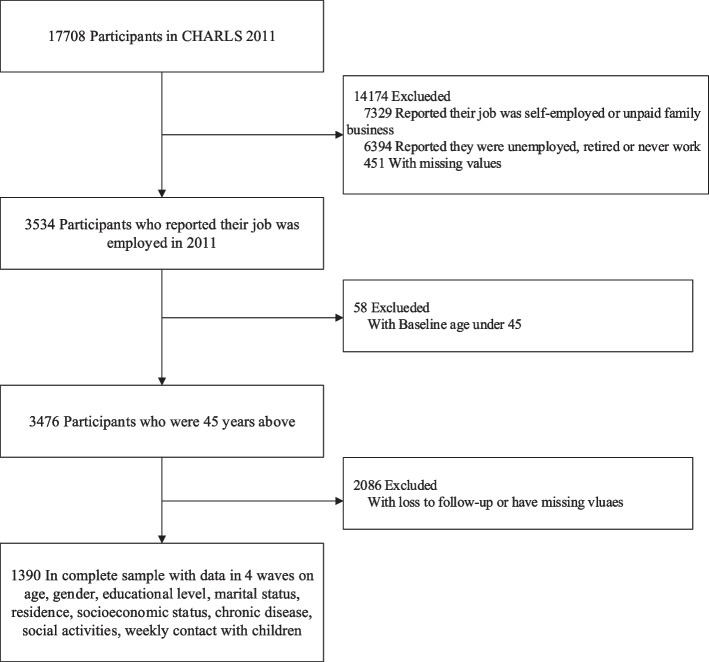
Flow chart of the participants

For this study, we included cases that were not lost in the four follow-ups and had no missing values in the independent or dependent variables. Participants’ work status was determined by a series of questions asked by CHARLS, including agricultural employed, agricultural self-employed, non-agricultural employed, non-agricultural self-employed, nonagricultural unpaid family business, unemployed, retired, and never worked. In the end, a total of 1390 participants over the age of 45 who were not retired at the time of the baseline survey and whose working status were employed (non-agricultural employed or agricultural employed) were included in the study (Fig. 1). CHARLS asks each respondent at series of questions to determine their work status. At wave 2 to 4, a respondent was labeled as retired if he or she was not currently engaged in agricultural or non-agricultural work but has worked for at least three months during their lifetime and has not searched for a job during the past month at the time of interview.

### Assessment of depressive symptoms

In this study, a ten-item Center for Epidemiologic Studies Depression (CES-D) scale, which has been validated among Chinese older adults was used to measure depressive symptoms [11]. Participants were asked to rate how often they experienced depressive symptoms in the past week from 0 (rarely or none of the time [< 1 day]) to three (most or all of the time [5–7 days]) [12]. The total score ranged from 0 to 30, and a lower score suggests lower level of depressive symptoms. Based on previous studies, a cut-off point of 12 is valid in identifying clinically significant depression [13]. Participants who got more than 12 points were considered to have depression symptoms.

### Assessment of covariates

Some potential confounders were considered in the analysis, which included age (1 = 45–49 years; 2 = 50–54 years; 3 = 55–59 years; 4 = 60–64 years; 5 = 65 plus), gender (1 = male; 2 = female), marital status (1 = married; 2 = unmarried and others), education level (1 = primary school and below; 2 = secondary school and above), residence (1 = rural; 2 = urban), employment type (1 = agricultural employed; 2 = non-agricultural employed), chronic disease (1 = yes; 0 = no). In addition, four socioeconomic level groups on the basis of quartiles of per-capita household consumption expenditure were defined, in which Q1 represents the lowest and Q4 represents the highest. Participates in social activities was assessed by self-reported participation in social activities a month before the interview, which was assigned 0 if the respondent did not participate in any of the social activities and assigned 1 if the respondent participated in at least one of the following social activities: interacted with friends; played Ma-jong, played chess, played cards, or went to community club; went to a sport, social, or other kind of club; took part in a community-related organization; done voluntary or charity work; attended an educational or training course. The variable contact with children was assigned 1 if the participants weekly contact with children in person or by phone, text message or email, and 0 otherwise.

### Statistical analysis

A panel data approach of random-effects logistic regression was used to examine the associations between retirement and depressive symptoms. The models were adjusted for age, gender, education level, marital status, residence and socioeconomic status, chronic disease, participation in social activities and contact with children. Sub group analysis were further performed to assess the differential effect in population groups. It was stratified by the same covariates above and the same regression analyses was used but with the stratification variable removed. For the logistic regression analyses, we report associations as odds ratios (ORs) with 95% CIs.

All descriptive analyses were weighted to account for the complex, multistage design of the study, and nonresponse in the CHARLS data. The regression analyses in our study were unweighted because previous studies using CHARLS data suggested that results from regression analyses with and without weighting are similar [12, 14]. The p values of less than 0·05 was considered to be significant.

## Results

Overall 1390 CHARLS cases (5560 records) over 45 years old who were employed in 2011 were include in this study. Eligible participants’ socio-demographic and socioeconomic characteristics are shown in Table 1. The median age of participants in 2011 was 53 years (IQR 48 ~ 58). 896 (64.46%) participants were male and 494 (35.54%) were female. 1251 (90%) participants were married, and 701 (50.43%) had secondary education and above. 796 (57.27%) were residing in rural areas, and 814 (58.56) reported have chronic disease. 693 (49.86%) participants reported that they participated in social activities in the past month at interview. Most participants (91.94%) had weekly contact with their children in person or by phone, text message, mail or email. There were 246 (17.7%) participants reporting CES-D scores of at least 12 in 2011, and the rate was increased to 23.81 percent in 2018 (Table S1).

**Table 1:** Scio-demographic characteristics of study participants, in 2011

Characteristics	Unweighted results				Weighted results		
	N	% of all	95%CI		% of all	95%CI	
All participants	1390	—	—	—	—	—	—
Age (years)							
45to49	479	34.46	31.96	37.03	36.91	36.9	36.92
50to54	320	23.02	20.83	25.33	24.48	24.47	24.49
55to59	346	24.89	22.64	27.25	22.88	22.87	22.89
60to64	166	11.94	10.28	13.76	10.63	10.62	10.64
65plus	79	5.68	4.53	7.03	5.1	5.09	5.1
Gender							
Male	896	64.46	61.88	66.98	60.55	60.54	60.57
Female	494	35.54	33.02	38.12	39.45	39.43	39.46
Education level							
Primary school and below	689	49.57	46.91	52.23	46.94	46.93	46.96
Secondary school and above	701	50.43	47.77	53.09	53.06	53.04	53.07
Marital status							
Married	1251	90	88.3	91.53	90.23	90.23	90.24
Unmarried and others	139	10	8.47	11.7	9.77	9.76	9.77
Residence							
Rural	796	57.27	54.62	59.89	49.41	49.4	49.42
Urban	594	42.73	40.11	45.38	50.59	50.58	50.6
Socioeconomic status							
Q1	348	25.04	22.78	27.4	21.45	21.44	21.46
Q2	347	24.96	22.71	27.33	22.79	22.78	22.8
Q3	349	25.11	22.85	27.47	26.2	26.19	26.22
Q4	346	24.89	22.64	27.25	29.56	29.55	29.57
Employment type							
Agricultural	285	20.5	18.41	22.72	17.92	17.91	17.93
Non-agricultural	1105	79.5	77.28	81.59	82.08	82.07	82.09
Chronic disease							
Yes	814	58.56	55.92	61.17	57.26	57.25	57.28
No	576	41.44	38.83	44.08	42.74	42.72	42.75
Participant in social activities							
Yes	693	49.86	47.19	52.52	51.01	51	51.02
No	697	50.14	47.48	52.81	48.99	48.98	49
Weekly Contact with Children							
Yes	1278	91.94	90.39	93.32	92.82	92.81	92.83
No	112	8.06	6.68	9.61	7.18	7.17	7.19

Table 2 present the results of random-effect logistic regression model evaluating the association between depressive symptoms risk and retirement. In all three models, participants who had retired were more likely to report depressive symptoms than those who were still working. In Model 1, people over 60 years of age have a higher risk of depression than the younger group. However, when chronic disease was added to the control variables (model 2 and 3), the effect of age became insignificant. Men and those with higher education level reported a lower risk of depression than women and people with lower education level. People whose marital status is unmarried and others are more likely to experience depression. The results above are consistent across all three models. The socioeconomic level is negatively correlated with the risk of depression. Compared with people employed in agricultural jobs, a lower risk of depression was found in people employed in non-agricultural jobs, and this result was consistent across the three models. People with chronic diseases were at higher risk of depressive symptoms than those without chronic diseases, and the OR values in model 2 and model 3 were 2.44 (95%CI 1.89–3.15) and 2.45 (95%CI 1.91–3.18), respectively. The result of model 3 also suggests that there is a negative correlation between participation in social activities and depression (adjusted OR 0.77, 95%CI 10.63–0.94).

**Table 2:** Longitudinal analysis of determinants of depressive symptoms among employed people aged 45 years and older in China, 2011–18

Variables		Adjusted odds ratio (95% CI)		
		**Model 1**	**Model 2**	**Model 3**
Retired	No	1.00 (ref)	1.00 (ref)	1.00 (ref)
	Yes	1.62 (1.23, 2.14) ***	1.52 (1.15, 1.99) **	1.5 (1.14, 1.97) **
Age (years)	45to49	1.00 (ref)	1.00 (ref)	1.00 (ref)
	50to54	1.32 (0.98, 1.77)	1.2 (0.89, 1.61)	1.22 (0.91, 1.64)
	55to59	1.35 (0.97, 1.89)	1.16 (0.83, 1.63)	1.18 (0.85, 1.66)
	60to64	1.62 (1.11, 2.35) *	1.32 (0.91, 1.91)	1.32 (0.91, 1.91)
	65plus	1.68 (1.09, 2.59) *	1.32 (0.86, 2.04)	1.33 (0.86, 2.04)
Gender	Female	1.00 (ref)	1.00 (ref)	1.00 (ref)
	Male	0.44 (0.33, 0.59) ***	0.44 (0.33, 0.59) ***	0.45 (0.34, 0.59) ***
Education level	Primary school and below	1.00 (ref)	1.00 (ref)	1.00 (ref)
	Secondary school and above	0.55 (0.41, 0.74) ***	0.57 (0.43, 0.75) ***	0.58 (0.44, 0.77) ***
Marital status	Married	1.00 (ref)	1.00 (ref)	1.00 (ref)
	Unmarried and others	1.73 (1.27, 2.35) ***	1.79 (1.32, 2.43) ***	1.74 (1.28, 2.35) ***
Residence	Urban	1.00 (ref)	1.00 (ref)	1.00 (ref)
	Rural	1.33 (0.99, 1.78)	1.36 (1.02, 1.81) *	1.27 (0.96, 1.69)
Socioeconomic status	Q1 (lowest)	1.00 (ref)	1.00 (ref)	1.00 (ref)
	Q2	0.79 (0.55, 1.14)	0.81 (0.57, 1.16)	0.79 (0.56, 1.13)
	Q3	0.77 (0.53, 1.13)	0.74 (0.51, 1.07)	0.76 (0.52, 1.09)
	Q4 (highest)	0.67 (0.46, 0.99) *	0.64 (0.44, 0.93) *	0.66 (0.45, 0.96) *
Employment type	Agricultural	1.00 (ref)	1.00 (ref)	1.00 (ref)
	Non-agricultural	0.51 (0.37, 0.71) ***	0.52 (0.38, 0.72) ***	0.5 (0.37, 0.69) ***
Chronic disease	No		1.00 (ref)	1.00 (ref)
	Yes		2.44 (1.89, 3.15) ***	2.46 (1.91, 3.18) ***
Participant in social activities	No			1.00 (ref)
	Yes			0.76 (0.62, 0.92) **
Weekly Contact with Children	No			1.00 (ref)
	Yes			0.78 (0.57, 1.07)

Model:1 Adjusted age, gender, education level, marital status, residence and socioeconomic status; Model 2: Model 1 plus adjustment for chronic disease; Model 3: Model 2 plus adjustment for participation in social activities and contact with children

^*^:*P* < 0.05; **: *P* < 0.01; ***:*P* < 0.001

**Fig. 2:**
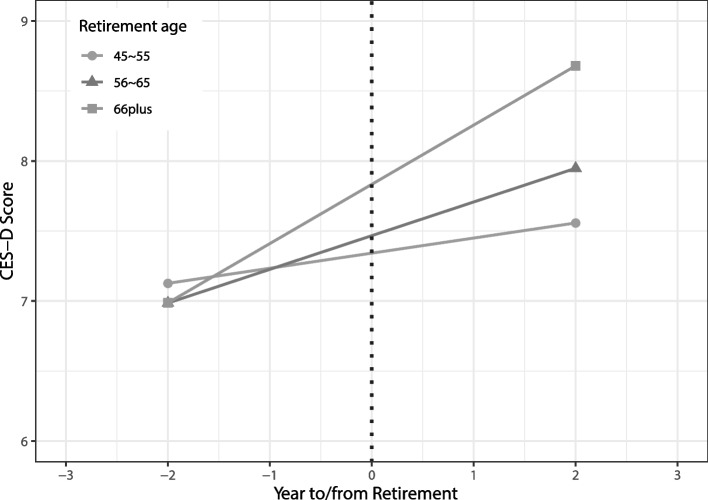
The CES-D score of employees in the years before and after retirement in different retirement age groups

**Fig. 3:**
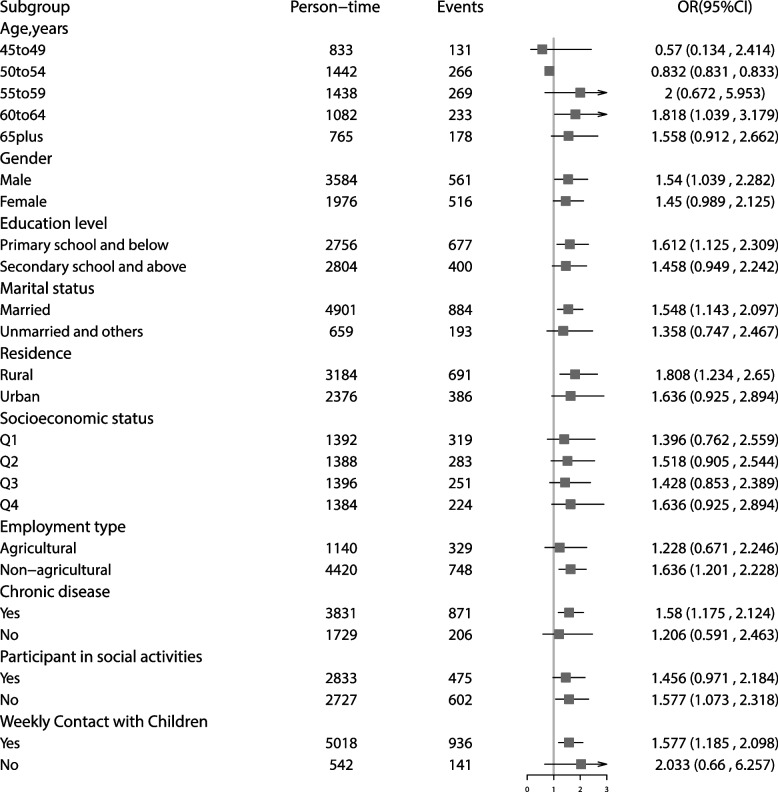
Adjusted odds ratios and 95% CI of retirement for depression in subgroups stratified by socio-demographic factors, employment type, chronic diseases and social activities. Effects are estimated for retirement on depression risk compared with unretired in each subgroup

Figure 2 shows the trend of the CES-D score of people in different retirement age groups in the years before and after retirement. The depression score during retirement increases to different levels in different groups, that the older at retirement period, the greater the increase. Figure 3 shows the relationship between retirement and depression in subgroups stratified by different age, gender, marital status, education level, place of residence, socioeconomic level, type of employment, whether to participate in social activities, and whether to contact their children weekly. The older subgroup had a higher risk of depression after retirement than the younger subgroup. The effects of retirement on depression was significant for males, individuals who married and with lower education level and living in rural areas. Compared with people without chronic diseases, people with chronic diseases were more susceptible to the impact of retirement. When stratified by employment type, increasing depression risk was found for non-agricultural employees. Retirees who did not participate in social activities were more likely suffer from depression. The relationship between retirement and depression is not significant among people with different socioeconomic levels. Figure S1 shows the pathway diagram of the influence of retirement and related covariates on depression.

Figure S2 shows the moderating effect of individual socioeconomic level, chronic disease status, and contact with children on the effect of retirement on depression. As can be seen from the figure, the prevalence of depression is significantly higher among retired people with low socioeconomic level, chronic diseases and less contact with their children.

## Discussion

In this study, we used national representative panel data to analyze the relationship between retirement and depressive symptoms in employees. We found that among employees, the prevalence of depression is about 20 percent. And the results showed that retirement would increase the risk of depression for the employees, the effect of which still exist after controlling for socio-demographic and socio-economic variables. For different subgroups stratified by socio-demographic factors, chronic diseases, and social activities, the effects of retirement on depression vary. The risk of depression increased among female, unmarried, living in rural areas, with lower education level, with lower socioeconomic status, not participants in social activities and with at least one chronic disease.

The prevalence of depression in this study is lower than other studies in China [12, 15, 16]. This may be due to the fact that in order to explore the effect of retirement on depression, the target person in this study excluded self-employed and non-working population. Regarding the psychological impact of retirement, the results vary in different countries and regions. Our research results were consistent with the results of Shiba et al., who found that both male and female retirements in Japan led to an increase in depression [17]. Park’s work showed that retirement increased the risk of depression after analyzing the 6-year follow-up data of 6,706 Koreans [18]. The result is similar to that of Noh et al., that retirement could worsen depressive symptoms. Bonilla-Tinoco suggested that the relationship between retirement and depression depends on the role of the social environment [19]. Olesen et al. analyzed the data of Danish workers and found that the prevalence of hospital treatment for depression showed an upward trend before and during retirement [2].

However, some researchers have reached diametrically opposite conclusions. Matta et al. analyzed the employees of the French national gas and electricity company and found that there was a main effect of retirement with a sharp decrease of depressive symptoms [20]. In the analysis of British civil servants, Fleischmann also found that retirement is beneficial to mental health: within three years after retirement, mental health has improved significantly and maintained for a long time [21]. Coursolle et al. conducted a follow-up study on American high school graduates and found that retirement may come more as a relief than as a stressor for individuals previously experiencing high levels of work demands interfering with family life. The findings of Belloni, et al. showed that retirement has a positive effect on men’s mental health, especially in areas severely affected by the economic crisis [22]. There are few Chinese studies on the relationship between retirement and depression. Zang et al. used CHALS database analysis to find that retirement is a protective factor for depression, but their research only includes people who are married and live in urban areas without chronic diseases [23].

Through subgroup analysis, we found that among people with different socio-demographic and socioeconomic levels, the effect of retirement on depression varies from person to person. The older the age, the greater the increase in depression caused by retirement. This may also be related to the health status of retirees. In our regression model, when chronic diseases are controlled, the effect of age on depression is no longer statistically significant. Among people with chronic diseases, retirement has a stronger effect on depression. Health status is not only directly related to depression, but in the transitional phase of retirement, older people or those with chronic diseases are more stressed. Moreover, Wang et al. found that retirees who were younger or had better health were more likely to engage in bridge employment than in full retirement [24]. For gender, we found that men seem to be more susceptible to the impact of retirement than women, which is similar to the results of related studies, that the relationship between retirement status and CES-D10 score gain was significantly in males but females [25]. This may be due to the traditional concept of “men outside the home, women inside” in the Chinese family. After retirement, men no longer work, and their social activities at work have been drastically reduced, so that they do not adapt to the changes in daily household chores and social networks in the family. On the contrary, women have always been responsible for daily household chores, grocery shopping, shopping, etc., and it is easier to adapt to the lifestyle after retirement [26].

People with lower education levels have significantly increased depression after retirement, which was consistent with the results of Assil et al. [27] and Wang et al. [9], that lower education levels were more likely to be depressed, and highly educated people typically have more capacity and options in maintaining their life patterns because of their professional knowledge and/or skills. People who are unmarried or who live in rural areas are more likely to experience depression after retirement. Mechakra found that people without a partner tended to consult for depression more frequently than people with a cohabiting partner [28]. Compared with rural areas, urban areas have higher pension coverage and more social activities, which are all factors that improve mental health [23].

Our results showed that even if a higher socioeconomic level was a protective factor for depression, retirement still led to a certain increase in the risk of depression among people with different socioeconomic levels; Similarly, although non-agricultural work was a protective factor for depression, when non-agricultural workers retire, there was a statistically significant increase in the risk of depression. This may be because non-agricultural jobs are relatively more stable than agricultural jobs, with regular working hours and higher incomes. After retirement, the protective factors of work will be removed, and individuals will face a larger gap [2].

Participation in social activities is a protective factor for depression. We found that retirees who did not participate in social activities had a higher risk of depression. Participating in substitute activities may provide similar benefits to work, and is more relevant to psychological well-being during the retirement phase of life [29].

Socioeconomic status, chronic disease, and contact with children moderated the effect of retirement on depression. The sharp drop in income after retirement reduces the ability of retirees to deal with various problems in life, especially health problems. Older people often accompanied by chronic diseases and need to obtain more medical resources. However, when the income level drops, the management of chronic diseases will be affected [14]. In addition, the change of roles from a relatively busy and regular lifestyle to a relatively inactive one might be a trigger for some retirees to develop mental health problems [30]. In recent years, young people live under great pressure and often ignore the contact with the old. Retirement might lead to social isolation, increasing the risk of depression [30].

Although it is imperative to raise retirement age in order to cope with the social problem of an aging population, appropriate protective measures must be taken to reduce the risk of depression in older people [31]. First, taking into account the moderating effect of the socio-economic situation, the policy of raising retirement age should be implemented in conjunction with the pension insurance system to protect the financial resources of retirees [31]. Second, older people need to be encouraged to participate in social activities, not only because these activities contain health-promoting components, but are congruent with societal expectations about the behaviors of retired people [29]. The government or employers can integrate information about the activities into their retirement plans and strengthen publicity at the same time [29, 32]. From an individual point of view, older people should be encouraged to participate more in family life before retirement, in order to promote adaptation to role changes and reduce the negative impact of retirement [26].The whole society should carry forward the traditional virtues of the Chinese nation and encourage young children to accompany the elderly more often. In addition, based on the fact that retirees’ health status is closely related to depression, continuing to improve the implementation and effect of the combination of medical and health care is needed.

Our research had some limitations. First, it was an observational study, and causal inference cannot be made. Besides, although many confounding factors are controlled, there are still many residual confounding that cannot be fully controlled. Second, the variables used in the study were self-evaluated by participants (such as retirement status), which may result in some biases. Third, due to the limited sample size, the interaction between retirement and other socio-demographic variables still needs to be further explored in the future.

## Conclusions

In summary, this study found that retirement will increase the risk of depression among employees. People who are male, with lower education level, married, living in rural areas, suffering from chronic diseases, and those who do not participate in social activities are more likely to experience depression after retirement. Under the implementation of the delayed retirement policy, there should be corresponding auxiliary measures to improve the mental health of older people.

## Supplementary Information

**Additional file 1:** **Table S1.** Prevalence of depressive symptoms of employed people by socio-demographic group, in 2011, 2013, 2015 and 2018.

**Additional file 2:** **Figure S1.** Pathway of retirement and related confounding variables on depression. **Figure S2.** Prevalence of depression by work status and socioeconomic levels(a), chronic disease(b), and contact with children(c), respectively.

## Data Availability

Data is publicly available. See: http://charls.pku.edu.cn/.

## References

[1] Xinhua. China's population aging deepens with 18.7% aged 60 or above. http://english.www.gov.cn/archive/statistics/202105/11/content_WS6099f574c6d0df57f98d953b.html. (Accessed (2021, December 19)).

[2] Olesen K, Rod NH, Madsen IE, Bonde JP, Rugulies R (2015). Does retirement reduce the risk of mental disorders? A national registry-linkage study of treatment for mental disorders before and after retirement of 245,082 Danish residents. Occup Environ Med.

[3] Alexopoulos GS (2005). Depression in the elderly. Lancet.

[4] Peristera P, Platts LG, Magnusson HL, Westerlund H (2020). A comparison of the B-spline group-based trajectory model with the polynomial group-based trajectory model for identifying trajectories of depressive symptoms around old-age retirement. Aging Ment Health.

[5] Ma Y, Liang C, Gu D, Zhao S, Yang X, Wang X (2021). How Social Media Use at Work Affects Improvement of Older People's Willingness to Delay Retirement During Transfer From Demographic Bonus to Health Bonus: Causal Relationship Empirical Study. J Med Internet Res.

[6] Michael I (2014). The Health Consequences of Retirement. J Hum Resour.

[7] Eibich P (2015). Understanding the effect of retirement on health: Mechanisms and heterogeneity. J Health Econ.

[8] Behncke S (2012). Does retirement trigger ill health?. Health Econ.

[9] Wang M, Shi J (2014). Psychological research on retirement. Annu Rev Psychol.

[10] Zhao Y, Hu Y, Smith JP, Strauss J, Yang G (2014). Cohort profile: the China Health and Retirement Longitudinal Study (CHARLS). Int J Epidemiol.

[11] Chen H, Mui AC (2014). Factorial validity of the Center for Epidemiologic Studies Depression Scale short form in older population in China. Int Psychogeriatr.

[12] Zhang Z, He P, Liu M, Zhou C, Liu C, Li H (2021). Association of Depressive Symptoms with Rapid Kidney Function Decline in Adults with Normal Kidney Function. Clin J Am Soc Nephrol.

[13] Wang R, Chen Z, Zhou Y, Shen L, Zhang Z, Wu X (2019). Melancholy or mahjong? Diversity, frequency, type, and rural-urban divide of social participation and depression in middle- and old-aged Chinese: A fixed-effects analysis. Soc Sci Med.

[14] Zhao Y, Atun R, Oldenburg B, McPake B, Tang S, Mercer SW (2020). Physical multimorbidity, health service use, and catastrophic health expenditure by socioeconomic groups in China: an analysis of population-based panel data. Lancet Glob Health.

[15] Yao Y, Lu T, Liu Y, Qin Q, Jiang J, Xiang H. Association of depressive symptoms with ambient PM(2.5) in middle-aged and elderly Chinese adults: A cross-sectional study from the China health and Retirement Longitudinal Study wave 4. Environ Res. 2021;203:111889. 10.1016/j.envres.2021.111889 34418451

[16] Wang Q (2020). Association of Childhood Intrafamilial Aggression and Childhood Peer Bullying With Adult Depressive Symptoms in China. JAMA Netw Open.

[17] Shiba K, Kondo N, Kondo K, Kawachi I (2017). Retirement and mental health: dose social participation mitigate the association? A fixed-effects longitudinal analysis. BMC Public Health.

[18] Park H, Kang MY (2016). Effects of voluntary/involuntary retirement on their own and spouses' depressive symptoms. Compr Psychiatry.

[19] Bonilla-Tinoco LJ, Fernández-Niño JA, Manrique-Espinoza BS, Romero-Martínez M, Sosa AL (2019). Retirement and depression in Mexican older adults: effect modifiers in a cohort based on the Study on AGEing and Adult Health (SAGE), 2002–2010. J Popul Ageing.

[20] Matta J, Carette C, Zins M, Goldberg M, Lemogne C, Czernichow S (2020). Obesity moderates the benefit of retirement on health: A 21-year prospective study in the GAZEL cohort. J Psychosom Res.

[21] Fleischmann M, Xue B, Head J (2020). Mental Health Before and After Retirement-Assessing the Relevance of Psychosocial Working Conditions: The Whitehall II Prospective Study of British Civil Servants. J Gerontol B Psychol Sci Soc Sci.

[22] Belloni M, Meschi E, Pasini G (2016). The Effect on Mental Health of Retiring During the Economic Crisis. Health Econ.

[23] Zang E (2020). Spillover effects of a husband's retirement on a woman's health: Evidence from urban China. Soc Sci Med.

[24] Wang M, Zhan Y, Liu S, Shultz KS (2008). Antecedents of bridge employment: a longitudinal investigation. J Appl Psychol.

[25] Noh JW, Kwon YD, Lee LJ, Oh IH, Kim J (2019). Gender differences in the impact of retirement on depressive symptoms among middle-aged and older adults: A propensity score matching approach. PLoS ONE.

[26] Chao Z, Xinjun W. The impact of retirement on residents' health—based on regression discontinuity design. Res Econ Manag. 2020;41:112–28.

[27] Assil SM, Zeidan ZA (2013). Prevalence of depression and associated factors among elderly sudanese: a household survey in Khartoum State. East Mediterr Health J.

[28] Mechakra-Tahiri SD, Zunzunegui MV, Dubé M, Préville M (2011). Associations of social relationships with consultation for symptoms of depression: a community study of depression in older men and women in Québec. Psychol Rep.

[29] Olesen SC, Berry HL (2011). Community participation and mental health during retirement in community sample of Australians. Aging Ment Health.

[30] Li W, Ye X, Zhu D, He P (2021). The Longitudinal Association Between Retirement and Depression: A Systematic Review and Meta-Analysis. Am J Epidemiol.

[31] Feng Y, Wang Q (2021). On the Imperative of the Overall Reform of China's Retirement System. J Northeast Normal Univ (Philos Soc Sci).

[32] Kaskie B, Imhof S, Cavanaugh J, Culp K (2008). Civic engagement as a retirement role for aging Americans. Gerontologist.

