# Supplementary material for: Association of depressive symptoms with retirement in Chinese employees: evidence from national longitudinal surveys from 2011 to 2018
Source: BMC Public Health. 2023 May 26;23:961. doi: 10.1186/s12889-023-15971-7 (PMC10214712; doi:10.1186/s12889-023-15971-7)
Supplement: Supplementary file 1 — Additional file 1: Table S1. Prevalence of depressive symptoms of employed people by socio-demographic group, in 2011, 2013, 2015 and 2018. [file 12889_2023_15971_MOESM1_ESM.docx]

**Table S1 Prevalence of depressive symptoms of employed people by socio-demographic group, in 2011, 2013, 2015 and 2018**

| **Characteristics** | **2011** | | | | **2013** | | | | **2015** | | | | **2018** | | | |
| --- | --- | --- | --- | --- | --- | --- | --- | --- | --- | --- | --- | --- | --- | --- | --- | --- |
|  | N | No. of respondents with depressive symptoms (n) | Unweighted  % | Weighted % | N | No. of respondents with depressive symptoms (n) | Unweighted  % | Weighted % | N | No. of respondents with depressive symptoms (n) | Unweighted  % | Weighted % | N | No. of respondents with depressive symptoms (n) | Unweighted  % | Weighted % |
| Total | 1390 | 246 | 17.7 | 15.27 | 1390 | 233 | 16.76 | 14.8 | 1390 | 267 | 19.21 | 17.39 | 1390 | 331 | 23.81 | 22.76 |
| Age,years |  |  |  |  |  |  |  |  |  |  |  |  |  |  |  |  |
| 45to49 | 479 | 77 | 16.08 | 13.35 | 285 | 42 | 14.74 | 11.77 | 69 | 12 | 17.39 | 16.24 |  |  |  |  |
| 50to54 | 320 | 59 | 18.44 | 14.03 | 371 | 55 | 14.82 | 12.77 | 461 | 90 | 19.52 | 17.73 | 290 | 62 | 21.38 | 24.27 |
| 55to59 | 346 | 59 | 17.05 | 16.39 | 377 | 68 | 18.04 | 16.47 | 348 | 59 | 16.95 | 12.98 | 367 | 83 | 22.62 | 20.18 |
| 60to64 | 166 | 38 | 22.89 | 22.06 | 228 | 45 | 19.74 | 17.56 | 311 | 57 | 18.33 | 17.74 | 377 | 93 | 24.67 | 22.78 |
| 65plus | 79 | 13 | 16.46 | 15.98 | 129 | 23 | 17.83 | 19.25 | 201 | 49 | 24.38 | 25.34 | 356 | 93 | 26.12 | 24.31 |
| Gender |  |  |  |  |  |  |  |  |  |  |  |  |  |  |  |  |
| Male | 896 | 125 | 13.95 | 13.26 | 896 | 123 | 13.73 | 13.09 | 896 | 138 | 15.4 | 14.4 | 896 | 175 | 19.53 | 18.87 |
| Female | 494 | 121 | 24.49 | 18.36 | 494 | 110 | 22.27 | 17.42 | 494 | 129 | 26.11 | 21.98 | 494 | 156 | 31.58 | 28.72 |
| Education level |  |  |  |  |  |  |  |  |  |  |  |  |  |  |  |  |
| Primary school and below | 689 | 152 | 22.06 | 20.08 | 689 | 143 | 20.75 | 19.57 | 689 | 173 | 25.11 | 23.06 | 689 | 209 | 30.33 | 28.36 |
| Secondary school and above | 701 | 94 | 13.41 | 11.02 | 701 | 90 | 12.84 | 10.57 | 701 | 94 | 13.41 | 12.37 | 701 | 122 | 17.4 | 17.8 |
| Marital status |  |  |  |  |  |  |  |  |  |  |  |  |  |  |  |  |
| Married | 1251 | 200 | 15.99 | 13.46 | 1247 | 206 | 16.52 | 14.32 | 1223 | 221 | 18.07 | 16.26 | 1180 | 257 | 21.78 | 21.16 |
| Unmarried and others | 139 | 46 | 33.09 | 31.98 | 143 | 27 | 18.88 | 19.22 | 167 | 46 | 27.54 | 26.43 | 210 | 74 | 35.24 | 32.33 |
| Residence |  |  |  |  |  |  |  |  |  |  |  |  |  |  |  |  |
| Rural | 796 | 160 | 20.1 | 19.14 | 796 | 147 | 18.47 | 18.02 | 796 | 171 | 21.48 | 21.4 | 796 | 213 | 26.76 | 25.85 |
| Urban | 594 | 86 | 14.48 | 11.5 | 594 | 86 | 14.48 | 11.65 | 594 | 96 | 16.16 | 13.47 | 594 | 118 | 19.87 | 19.74 |
| Socioeconomic status |  |  |  |  |  |  |  |  |  |  |  |  |  |  |  |  |
| Q1 | 348 | 69 | 19.83 | 19.71 | 348 | 70 | 20.11 | 20.39 | 348 | 80 | 22.99 | 23.37 | 348 | 100 | 28.74 | 28.99 |
| Q2 | 347 | 70 | 20.17 | 19.25 | 347 | 73 | 21.04 | 20.61 | 347 | 67 | 19.31 | 17.87 | 347 | 73 | 21.04 | 18.88 |
| Q3 | 349 | 56 | 16.05 | 13.7 | 349 | 47 | 13.47 | 12.17 | 349 | 63 | 18.05 | 17.6 | 349 | 85 | 24.36 | 22.92 |
| Q4 | 346 | 51 | 14.74 | 10.38 | 346 | 43 | 12.43 | 8.58 | 346 | 57 | 16.47 | 12.48 | 346 | 73 | 21.1 | 21.09 |
| Chronic disease |  |  |  |  |  |  |  |  |  |  |  |  |  |  |  |  |
| Yes | 814 | 177 | 21.74 | 19.1 | 881 | 177 | 20.09 | 17.8 | 1038 | 227 | 21.87 | 19.45 | 1098 | 290 | 26.41 | 25.1 |
| No | 576 | 69 | 11.98 | 10.14 | 509 | 56 | 11 | 9.98 | 352 | 40 | 11.36 | 11.37 | 292 | 41 | 14.04 | 14.16 |
| Participant in social activities |  |  |  |  |  |  |  |  |  |  |  |  |  |  |  |  |
| Yes | 693 | 100 | 14.43 | 11.92 | 774 | 108 | 13.95 | 12.45 | 728 | 133 | 18.27 | 15.69 | 638 | 134 | 21 | 21.16 |
| No | 697 | 146 | 20.95 | 18.76 | 616 | 125 | 20.29 | 17.84 | 662 | 134 | 20.24 | 19.31 | 752 | 197 | 26.2 | 24.3 |
| Weekly Contact with Children |  |  |  |  |  |  |  |  |  |  |  |  |  |  |  |  |
| Yes | 1278 | 215 | 16.82 | 14.34 | 1243 | 201 | 16.17 | 14.06 | 1262 | 233 | 18.46 | 16.83 | 1235 | 287 | 23.24 | 22.21 |
| No | 112 | 31 | 27.68 | 27.35 | 147 | 32 | 21.77 | 21.46 | 128 | 34 | 26.56 | 22.59 | 155 | 44 | 28.39 | 27.73 |
