# Supplementary material for: Association of depressive symptoms with retirement in Chinese employees: evidence from national longitudinal surveys from 2011 to 2018
Source: BMC Public Health. 2023 May 26;23:961. doi: 10.1186/s12889-023-15971-7 (PMC10214712; doi:10.1186/s12889-023-15971-7)
Supplement: Supplementary file 2 — Additional file 2: Figure S1. Pathway of retirement and related confounding variables on depression. Figure S2. Prevalence of depression by work status and socioeconomic levels(a), chronic disease(b), and contact with children(c), respectively. [file 12889_2023_15971_MOESM2_ESM.pdf]

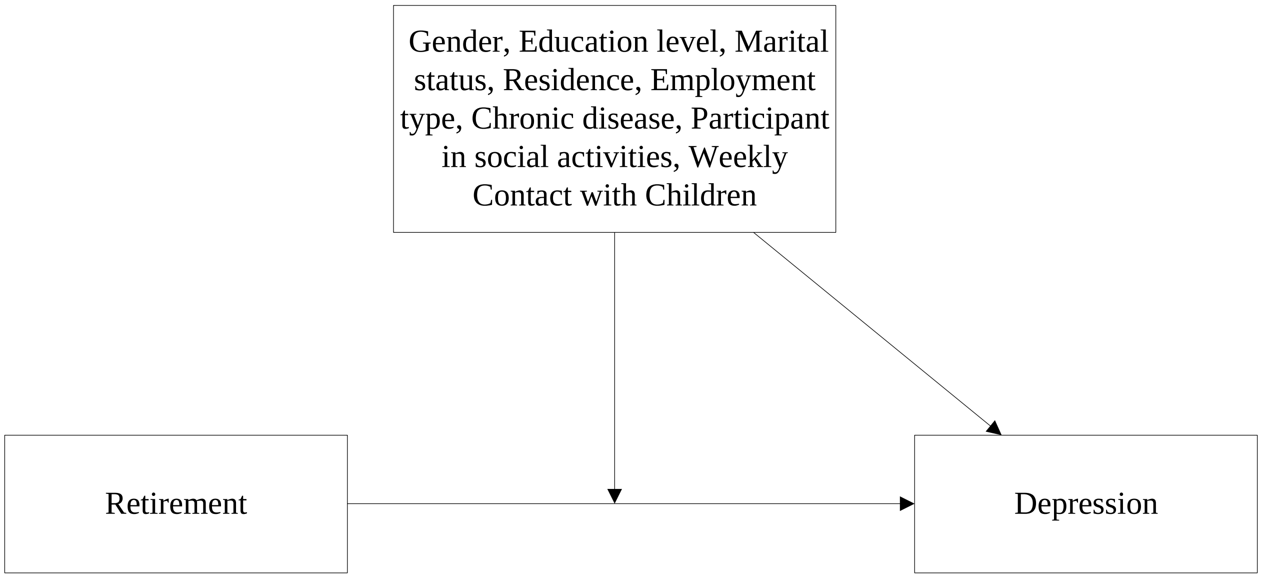

Figure S1 Pathway of retirement and related confounding variables on depression.

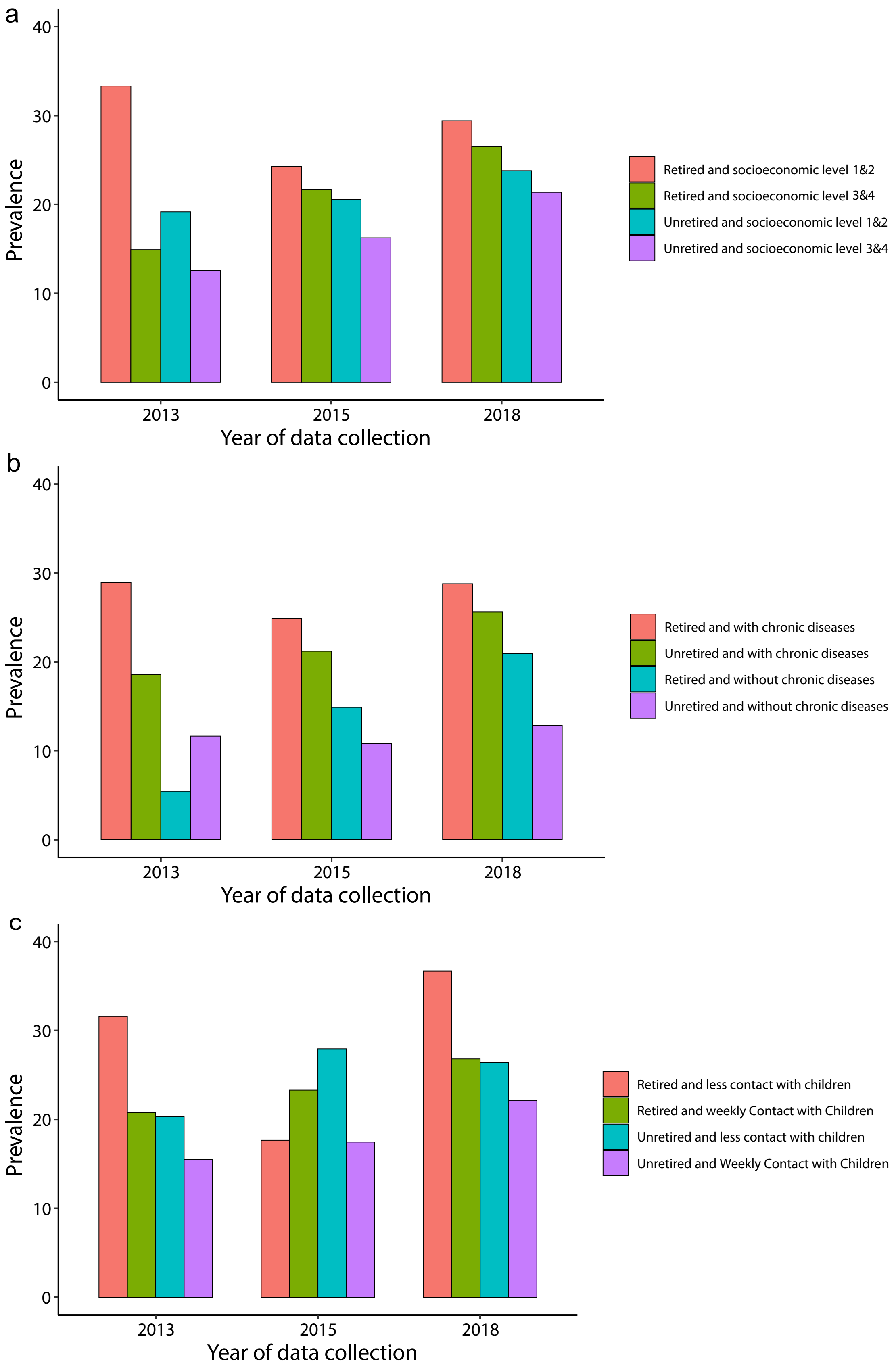

Figure S2 Prevalence of depression by work status and socioeconomic levels(a), chronic disease(b), and contact with children(c), respectively.
